# The quality of the organic materials determines its carbon conversion efficiency in tropical latosol

**DOI:** 10.3389/fmicb.2025.1573984

**Published:** 2025-05-01

**Authors:** Shuhui Song, Siru Liu, Yanan Liu, Lei Shi, Huayong Li, Weiqi Shi, Haiyang Ma

**Affiliations:** Key Laboratory of Tropical Crops Nutrition of Hainan Province, South Subtropical Crops Research Institute, Chinese Academy of Tropical Agricultural Sciences, Zhanjiang, Guangdong, China

**Keywords:** tropical organic materials, microbial community, carbon conversion efficiency, organic carbonization structure, latosol

## Abstract

**Introduction:**

Tropical regions are characterized by high temperatures and abundant rainfall, which facilitate rapid carbon mineralization. However, research on soil organic carbon conversion efficiency (Esoc) in these areas is currently constrained by a lack of robust data support.

**Methods:**

This study used nylon - bags with typical tropical organic materials (pineapple leaf (PAL), banana stems (BAS), coconut husk (CCH), and organic fertilizer (OF)) to explore how mixing straw with latosol impacts soil organic carbon conversion efficiency (Esoc) and products, and to understand the relationships among Esoc, material composition (glycolipid, hemicellulose, cellulose, lignin), and enzyme activity.

**Results:**

CCH had the highest Esoc, from 37.79% to 96.87%, followed by OF with 26.71%–63.12%. The Esoc of PAL and BAS was 34.57% and 25.32% at 90 days, and 7.59% and 2.55% at 1080 days. The main factor that determines the difference in carbon conversion efficiency is the composition of organic materials. Compared with CK treatment, the soil organic carbon for PAL and BAS at 90_days was mainly O–alkyl–C, anomertic–C, and N–alkyl/methoxyl–C, with an unstable structure. The decomposition products of CCH mainly consisted of anomertic–C, aromatic–C, O–alkyl–C, carbonyl–C, and N–alkyl/methoxyl–C. The increased organic carbon in OF - mixed soil was mainly N–alkyl/methoxyl–C and anomertic–C. In the short-term (90 days), PAL, BAS, and OF increased the quantity and diversity of soil microorganisms, as well as the activities of xylosidase and cellobiohydrolase. CCH mainly enhanced soil phenol oxidase activity and maintained microbial biomass stabilityin the long-term (1080 days).

**Discussion:**

This study revealed the changes of microbial diversity and enzyme activity under different organic materials. The promotion effects of PAL and BAS on microbial biomass, diversity and enzyme activity in the short term and the maintenance effects of CCH on the stability of microbial biomass in the later period were investigated, which provided a new basis for further exploring the function and mechanism of microorganisms in soil ecosystems.

## Highlights

- The main factor that determines the difference in carbon conversion efficiency is the composition of organic materials.
- CCH exhibited the highest Esoc, ranging from 37.79% to 96.87%, followed by OF with an Esoc of 26.71%–63.12%. In contrast, after 1080 days of cultivation, PAL and BAS had relatively lower Esoc values, at 7.59% and 2.55% respectively.
- PAL and BAS can increase microbial biomass, diversity and enzyme activity in the short-term, while CCH can maintain the stability of microbial biomass in the long-term.

## 1 Introduction

Soil organic carbon (SOC) is a key indicator of soil fertility and health (Liu et al., 2006; He et al., 2018). While, lateritic soils are very poor in SOC (Pramanik et al., 2010) owing to high temperature and rainfall and intense microbial activity in tropical and subtropical areas of China. Organic materials application to agricultural soils is widely used as a common management practice to increase soil SOC stock (He et al., 2018).

In South China, banana, pineapple, and coconut productions have considerable economic importance. After harvesting, pineapple leaves (PAL), and banana stems (BAS) are left over in the fields (Deng et al., 2021; Saini et al., 2023). The coconut husk (CCH) has various uses and mainly comprises cellulose, hemicellulose and other natural polymer substances that belong to the pure natural biomass medium, and a renewable biomass resources comprising potassium, calcium, nitrogen (N), phosphorus (P), magnesium and other nutrient elements (Deng et al., 2021). In general, these waste biomass are abundant natural resources in subtropical and tropical regions and can provide potential for providing profitable products such as manure (Ultra et al., 2005), feed (Ulloa et al., 2004) and substrate (Xu et al., 2017a). Therefore, exploitating of the waste biomass can be of considerable benefit to the environment and bring additional profits to farmers.

The retention of this waste biomass can replenish soil organic carbon (SOC), and increase the activity and quantity of microbes in the soil (Zhou et al., 2016; Han et al., 2020; Deng et al., 2021; Mi et al., 2023). However, the biomass retention may have a negative impact on the release of CO_2_ in tropical regions of China. Biomass decomposition determines the rate of CO_2_ release and the efficiency of soil carbon (C) conversion and organic C structure of the soil, and its decomposition can differ because of the biochemical and physical properties, such as, material composition, and biomass size (Makkonen et al., 2012; Erhagen et al., 2013; Ji et al., 2024). Thus, a thorough understanding of typical tropical organic material is a prerequisite to developing best management practices for using tropical biomass residues.

Different forms of biomass residue (direct straw return, converted as manure and pyrolyzed as biochar) affect soil organic carbon (SOC) pools and soil organic matter (SOM) composition (Sun et al., 2021; Chen et al., 2024). The structure and quantity of SOC are the key to maintaining the stability of the soil C pool, and microbial C use efficiency also plays a key role in regulating soil C flow (Domeignoz-Horta et al., 2020). The efficiency of soil C conversion may vary with the cellulose, hemicellulose, and lignin content of raw material, soil moisture, temperature and climates. For example, in mollisols, crop residue return could either cause an increase or decrease in the relative abundance of aromatic –C (Zheng et al., 2021; Hao et al., 2022). Changes in crop straw decomposition and C conversion efficiency (Esoc) will inevitably cause changes in decomposition products and the formation of SOC (Singh et al., 2005).

Esoc is closely linked to organic matter quality. Chemical composition, such as high levels of recalcitrant hemicellulose and lignin, can maintain Esoc stability, while a high carbon - to - nitrogen ratio generally impedes decomposition (Chen et al., 2019). Temperature affects microbial activity, with higher temperatures generally accelerating organic matter conversion, but extreme heat can be detrimental. Temperature also impacts soil moisture and aeration, which in turn influence Esoc (Davidson and Janssens, 2006; Fierer et al., 2003a). Soil properties, soil pH impacts the microbial community structure and chemical reactions related to organic matter, indirectly affecting Esoc (Rousk et al., 2010; Chen et al., 2019). Several studies have investigated the effects of crop straw placement depths on the straw decomposition rates (Giacomini et al., 2007; Curtin et al., 2008) and the chemical composition of straw residues (Cui et al., 2017) in the laboratory and field. However, fewer studies have sought to differentiate the effects of mixing tropical straw residues with latosol on Esoc and products in tropical regions of China.

The decomposition of straw residues is traditionally investigated by using litterbag methods in natural ecosystems (Jacobs et al., 2011; Xu et al., 2017b). As the SOM decomposition of is mediated by soil enzymes and microbial, detailed studies of these enzymes are central to understanding the decomposition of organic matter (Leinweber et al., 2008). The diversity of microbial communities and enzyme activities can be changed by different straw returns. The abundances of proteobacteria and bacteroidetes were increased, whereas the abundances of chlorobacteria, firmicutes and nitrospirulina were decreased (Zhang et al., 2024). Therefore, understanding the responses of the soil organic structure to different forms of crop residues could help explore the technology of soil organic carbon (SOC) stabilization enhancement. Enzymes are usually secreted by microorganisms, and enzyme activity usually serves as an indicator of microbial nutrient requirements and is often related to SOM components (Błońska et al., 2016). Several studies have attempted to link the activity of enzymes involved in C (Shi et al., 2006), N, and P cycling, with the molecular structure, turnover dynamics, and chemical composition of SOM, respectively (Orwin et al., 2006; Zak and Kling, 2006; Leinweber et al., 2008; Tian et al., 2010).

Tropical regions are characterized by high temperatures and abundant precipitation. In these areas, carbon (C) cycling occurs at a rapid pace. Paradoxically, despite the brisk C conversion, the utilization efficiency of organic materials remains low. Currently, there is a dearth of comprehensive and reliable data to support evaluations of the ecotoxicological effects (Esoc) of different organic materials in the tropical regions of China. Therefore, the following main objectives of this study were to understand: how the Esoc of typical tropical organic materials change in latosol; if tropical organic materials shifted the chemical composition and diversity of SOM; and what is the process and microbial mechanism of Esoc change after different organic materials applied to latosol. We hypothesized that the soil Esoc and chemical composition of SOC were differed by changing soil enzyme activity and microbial community diversity after different components of tropical typical organic materials were added into latosol.

## 2 Materials and methods

### 2.1 Site description and litter experiment in the field

The field experimental sites located at the National agricultural experimental station for soil quality, Zhanjiang (47°26′N, 126°38′E) in South China. The average annual temperature in this region is 22.7°C ~ 23.5°C, which belongs to the monsoon climate in the northern margin of the tropical. The average annual rainfall is 1395.5 ~ 1723.1 mm, and the average annual sunshine duration is 1714.8 ~ 2038.2 h. During the test period, the lowest temperature of the soil in the test field was 17.6°C, the highest temperature was 25.7°C, and the average temperature was 20.7°C. Sweet maize was planted in the previous crop, and corn was planted in the surrounding area except for the experimental area in the test year.

Organic materials were pineapple leaf (PAL), banana stems (BAS), coconut husk (CCH), and organic fertilizer (OF). Pineapple leaf (PAL) and banana stems (BAS) were collected from Experimental field of South Subtropical Crops Research Institute of Chinese Academy of Tropical Agricultural Sciences. Coconut husk (CCH), and organic fertilizer (OF) were commodity material. The materials were air-dried and chopped by a crop cutter into 1–2 cm pieces. The original materials were ground with a ball mill to measure total C and N contents by an element analyzer (Vario MACRO Cube, Elementar, Germany). The addition amount of C is 2% (C: soil). (NH_4_)_2_SO_4_ was used to adjust the origin C/N ratio with 23.

At site, soil samples of 0–20 cm deep were taken randomly from an arable land with sweet maize cropping. The soils samples were air-dried and ground to pass a 2-mm sieve for chemical and physical analysis. Soil pH in water (1: 2.5) was measured by a pH meter. The < 2 mm soil samples were further ground to pass through a 0.15-mm sieve to measure soil organic C and total N contents by an element analyzer. Basic physical and chemical soil properties before the experiment were as follows. pH: 5.74, EC: 40.30 *μ*s/cm, available P: 14.32 mg/kg, available K: 73.63 mg/kg, soil alkaline N: 88.54 mg/kg, Exchangeable Ca: 443.08 mg/kg, Exchangeable Mg: 75.09 mg/kg, available Mn: 33.92 mg/kg, available Fe: 15.00 mg/kg, available Cu: 1.50 mg/kg, available Zn: 0.87 mg/kg, C: 1.21%, N: 0.09%, δ^13^C: −20.79, δ^15^N: 6.98.

Nylon litterbags in the size of 10 cm long × 10 cm wide and having apertures of 200 mesh were used in this study. Such a mesh-size was considered to prevent soil particle exchanges but allow water and microbial exchanges between inside and outside the nets. The soil bulk density was as high as 1.0–1.1 g/cm^3^ like that of the surrounding soil. 250.0 g (dry basis) of soil and organic materials were filled into each nylon litterbag, and the amounts of organic materials were shown as Table 1. The litterbags were placed at 20 cm depth, and covered with soil. No crops were grown to avoid root disturbance in the plot (80 m^2^) where the litterbags were placed. Sixteen nylon litterbags per treatment were prepared to ensure 4 replicates at each of 4 sampling times during the observation period until the experiment end of November 2023.

**Table 1:** C, N content of organic materials and the amounts of organic materials in this experiment.

Organic materials	C content	N content	δ^13^C	δ^15^N	Amount	Amount of (NH_4_)_2_SO_4_
	%				g	
Pineapple Leaf (PAL)	41.79	0.57	−14.63	0.22	11.96	0.62
Banana stems (BAS)	37.78	0.34	−24.05	5.05	13.23	0.73
Coconut husk (CCH)	27.93	0.19	−27.42	4.18	17.90	0.78
Organic fertilizer (OF)	27.47	2.35	−21.09	6.12	18.20	0.00

### 2.2 Composition of organic materials

The hemicellulose, cellulose and lignin content were determined according to the method of Jung et al. (2015). Firstly, Extractive-free samples were prepared using the Soxhlet extraction method, and holocellulose from extractive-free samples was isolated using a delignification process. Secondly, acid-soluble lignin and alkali-soluble lignin were estimated according to the modified Klason lignin determination procedure and UV spectroscopic method. In this study, the lignin content is the sum of acid-soluble lignin and alkali-soluble lignin. The cellulose content in holocellulose was determined according to the method: KS M 7044. Hemicellulose weight was calculated by subtracting the weight of holocellulose from the weight of cellulose.

### 2.3 C and N content of soil

The elemental carbon (C), and nitrogen (N) were determined using an elemental analyzer (Elementar Vario UNICUBE, Elementar, Germany). Organic carbon content can be represented by total carbon content in latosol.


$$\mathrm{Esoc}\left(\%\right)=\frac{(\mathrm{SO}C_{\mathrm{sample}}-\mathrm{SO}C_{\mathrm{control})\;\times \;M_{\mathrm{soil}}}}{TOC_{\mathrm{straw}}\times M_{\mathrm{straw}}}\times 100\%$$

$$C\;\mathrm{loss percentage}\left(\%\right)=100\%-\mathrm{Esoc}$$

$$\Delta \;Esoc_{i}=Esoc_{t1}-{\mathrm{Esoc}}_{t2}$$

Where, SOC_sample_ was the C content of soil with mixing organic materials; SOC_control_ was the C content of soil without mixing organic materials; M_soil_ was the total weight of soil mixing with organic materials of each nylon litterbag; TOC_straw_ was the C content of organic materials; M_straw_ was the weight of organic materials in each nylon litterbag; i = 1, 2, or 3; t1, t2 is the continuous sampling time (90d, 270d, 540d, 1080d).

### 2.4 Microbial analysis

The soil microbial community composition was determined by a PLFA analysis (Wu et al., 2009). Briefly, the soil samples were freeze-dried, and then PLFAs were extracted with a single-phase mixture of chloroform: methanol: citrate buffer (1: 2: 0.8 volumetric ratios, pH 4.0). Neutral lipids and glycolipids were separated from polar lipids on a silica-bonded phase column (SPE-Si, Supelco, Poole, UK) by elution with chloroform and acetone, respectively. Nonadecanoic acid methylester (19:0) was added as the internal standard, and the polar lipids were converted to fatty acid methyl esters by a mild alkaline methanolysis. Dried fatty acid methyl esters were redissolved in n-hexane and then quantified and identified by gas chromatography (N6890, Agilent Technologies, Santa Clara, CA, USA) and MIDI Sherlock microbial identification system version 4.5 (MIDI Inc., Newark, DE, USA), respectively. The internal standard (19:0) peak was used as a reference to calculate the concentration of PLFAs, which was expressed as nmol/g dry soil.

Gram-positive (G^+^) bacteria were identified by the PLFAs: i13:0, i14:0, i15:0, a15:0, i16:0, a16:0, i17:0, a17:0, i18:0, i19:0, i20:0; and Gram-negative (G^−^) bacteria by the PLFAs: 15:1w6c, 16:1w5c, 16:1w7c, 16:1w9c, 17:1w8c, 18:1w5c, 18:1w6c, 18:1w7c, 18:1w9c, 20:1w9c, 16:02OH, cy17:0 and cy19:0 (Bach et al., 2010; Ma et al., 2015). The PLFA 18:1w9c, 18:2w6c, 18:3w6c, 18:3w3c, 18:2w6,9c, 20:1w9c were chosen to represent fungi; 16:1w5c to represent arbuscular mycorrhizae fungi (AMF); and 10Me 16:0, 10Me 17:0 and 10Me 18:0 to represent actinomycetes; 15:0, 17:0, i15:0, i16:0, i17:0, a15:0, a17:0 were used as aerobic bacteria, and cy17:0, cy19:0, 18:1w7c were used as biomarkers of anaerobic bacteria (Frostegard et al., 1993; Fierer et al., 2003b).

### 2.5 Soil-state ^13^C NMR spectroscopy

The soil from three replicates in each treatment were thoroughly mixed and sampled at each sampling time for analysis of chemical composition by using ^13^C cross polarization with total sideband suppression (^13^C CP/TOSS) NMR experiments without and with spectral-editing experiments (Han et al., 2020). In NMR spectroscopy studies, replicate analyses were not usual because of prohibitive costs. The ^13^C CP/TOSS NMR experiments were conducted with Bruker AVANCE 400 (Bruker Biospin, Rheinstetten, Germany) at 100 MHz for ^13^C with 4-mm sample rotors, at a spinning speed of 5 kHz, a CP time of 1-ms, a 1 H 90° pulse-length of 4-*μ*s, and a recycle delay of 0.8 s. Fourpulse total suppression of sidebands (TOSS) was employed before detection, and two-pulse phase-modulated decoupling was applied for optimum resolution. The ^13^C chemical shifts were referenced to the carbonyl signal (176.4 ppm) of glycine as an external standard. The ^13^C NMR signals in the CP/TOSS spectra were assigned into different C functional groups following the literature. The main assignments were as follows: alkyl–C (0–44 ppm), N–alkyl/methoxyl–C (44–68 ppm), O–alkyl–C (68–94 ppm), anomeric–C (94–113 ppm), aromatic–C (113–162 ppm) and carbonyl–C (162–220 ppm).

### 2.6 Statistics

Pearson linear correlations were employed on the data of Esoc, total C, total N, and the relative abundance of different C functional groups. Principal component analysis (PCA) of six main C functional groups expressed in the relative abundance derived from the NMR experiments, to determine the effects of mixing straw with soil. The statistics were performed using the SPSS 19.0 statistical software. All the plots were drawn using Origin 2021.

## 3 Results

### 3.1 Composition of organic materials

Different materials vary considerably in their composition. Organic fertilizer (OF) is the product of agricultural waste such as straw and manure after decomposition, fermentation and microbial decomposition, and the monosaccharide and proteins components account for >60% (Table 2). The content of monosaccharide/proteins did not considerably differ among the PAL, BAS, and CCH treatments, and the average content of glycolipids was 41.1%. The hemicellulose content in BAS and PAL accounted for >30%, which was considerably higher than that of CCH and OF. The lignin content in CCH was >25%, whereas the lignin content in BAS and OF was the lowest, with an average of only 1.7%. The ratio of sugar and lipid components to the sum of hemicellulose, cellulose and lignin in the materials of PAL, BAS and CCH was 1: 1.1, 1: 1.4, and 1: 1.4, with an average of 1: 1.3, respectively, whereas the ratio of OF was 1: 0.4. The OF considerably differed from PAL, BAS and CCH in terms of material components. The proportion of hemicellulose and cellulose + lignin in the PAL, BAS, and CCH was 1.4, 1.6, and 0.2, respectively, indicating that CCH was considerably different from PAL and BAS in this component.

**Table 2:** Composition of organic materials.

Species/component	Monosaccharide/proteins	Hemicellulose	Cellulose	Lignin
PAL	45.33 ± 9.00b	30.00 ± 6.61a	11.90 ± 3.00b	9.30 ± 0.26b
BAS	40.33 ± 0.58b	34.83 ± 2.84a	19.57 ± 3.33a	1.83 ± 0.25c
CCH	37.50 ± 1.32b	9.50 ± 0.50b	18.27 ± 0.25a	25.77 ± 0.75a
OF	62.50 ± 6.50a	11.17 ± 2.75b	12.43 ± 1.45b	1.67 ± 0.45c

Data are means ± standard error, *n* = 4. Different lowercase letters indicate significant differences (*p* < 0.05) among treatments. The same below.

### 3.2 C and N contents of latosol

**Figure 1:**
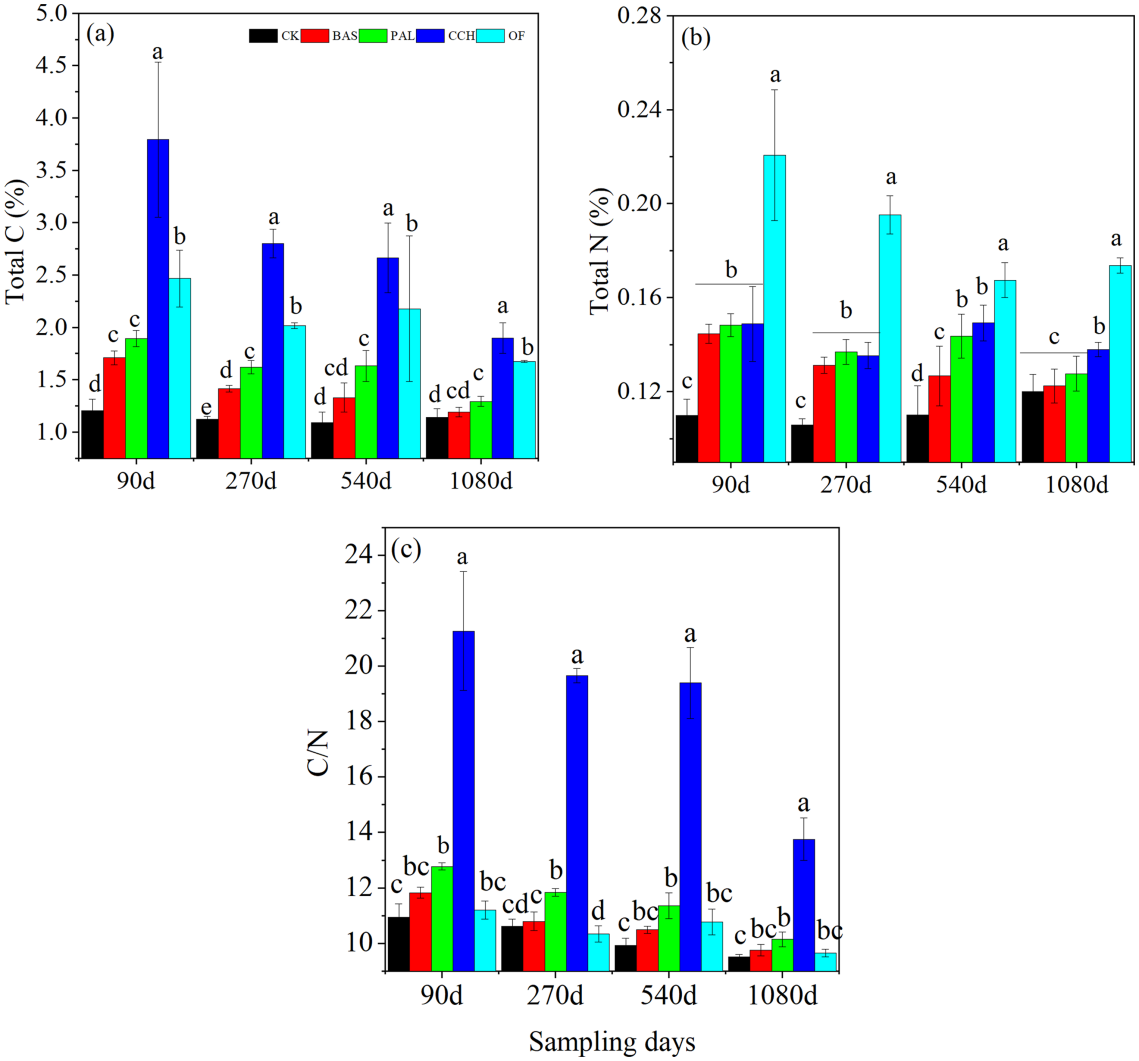
Total C content **(a)**, total N content **(b)**, and C/N ratio **(c)** of the organic materials-soil mixing. Different lowercase letters indicate significant differences (*p* < 0.05) among treatments. The same below. Vertical bars are standard errors (*n* = 4).

Total C and N contents and the C/N ratio of soil showed similar trends during the experimental period as the total C content and C/N ratio considerably decreased with time (Figures 1a–c). The total C content decreased from the 90-day value of 1.71–1.19% in BAS, 1.89–1.29% in PAL, 3.79–1.89% in CCH, and 2.46–1.67% in OF. For the different materials, the order of the C content among the treatments was CCH > OF > PAL > BAS > CK (Figure 1a). At 1080 days, the total C content in BAS and PAL was similar to that of CK. The C content was considerably affected by the addition of organic materials. The total N content decreased from 90 to 270 days and then increased slowly at 540 days, followed by a decrease at 1080 days, especially in CCH and PAL. The N content in CK increased from the value at 270 days of 0.11% to that at 1080 days of 0.12% (Figure 1b). This indicated that the N content was affected by the organic materials and other factors such as rainfall. The C/N ratio ranged from 9.51 to 21.26 during the sampling time. The C/N ratio of CK, BAS, PAL, and OF was similar with the trends of 10.94–9.51, 11.82–9.75, 12.78–10.14, and 11.19–9.65, respectively. The C/N ratio in CCH ranging from 21.26 to 13.75 was higher than that in other treatments. The C/N was affected by organic materials (Figure 1c and Table 3).

**Table 3:** Effects of mixing organic materials with soil, experiment times and their interactions on the total C, N and C/N, respectively, using a multifactorial ANOVA.

	Df	Total C (%)		Total N (%)		C/N		Carbon mineralization rate%	
		*F*	*p*	*F*	*p*	*F*	*p*	*F*	*p*
Date (D)	3	19.469	<0.0001	639.227	<0.0001	109.137	<0.0001	74.821	<0.0001
OMS	4	5.028	0.001	26.122	<0.0001	7.890	<0.0001	244.945	<0.0001
D × OMS	12	4.175	<0.0001	21.615	<0.0001	6.194	<0.0001	7.192	<0.0001

OMS, mixing organic materials with soil.

### 3.3 Carbon conversion efficiency

**Figure 2:**
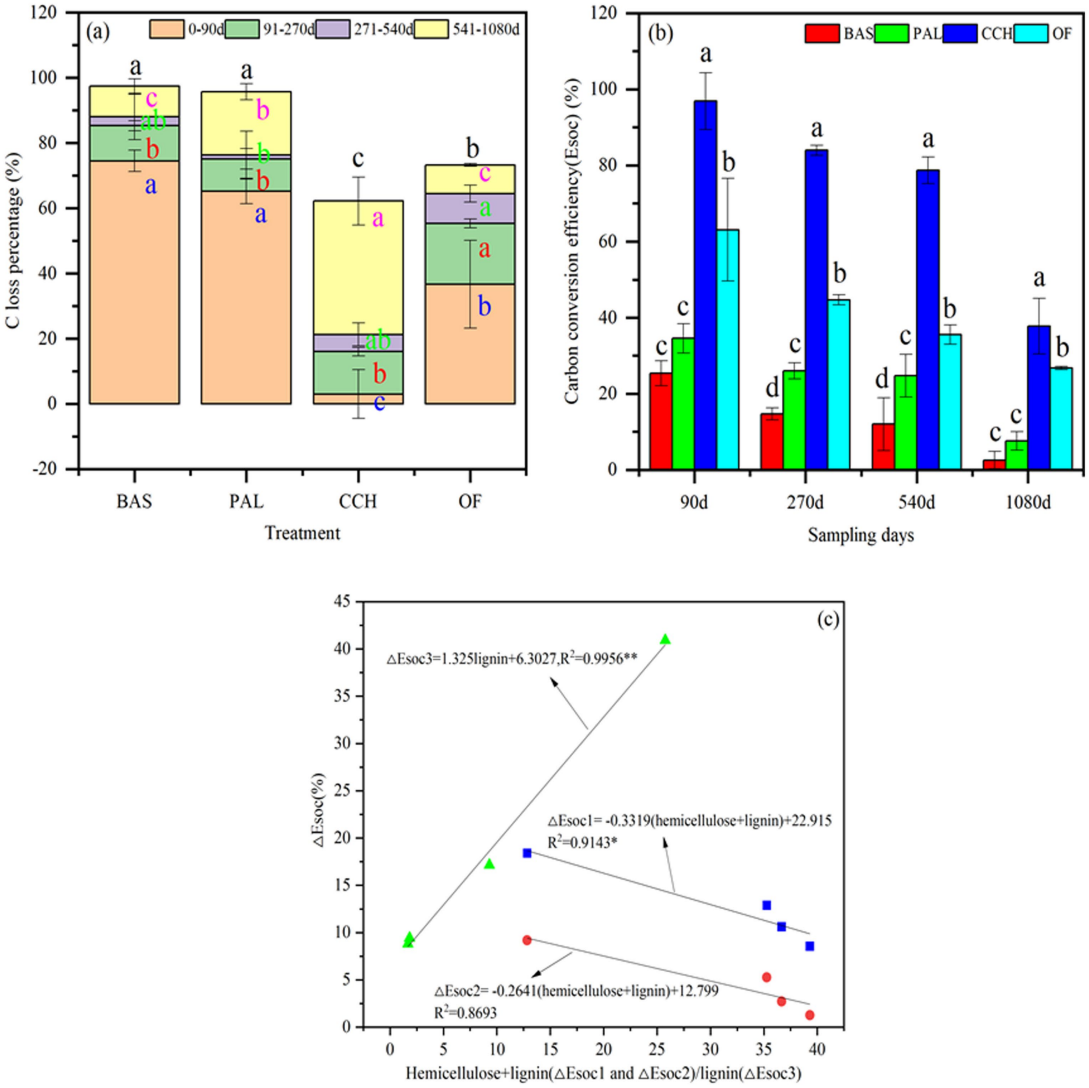
Carbon loss percentage **(a)**, Carbon conversion efficiency **(b)** and The relationship between ΔEsoc and material composition **(c)**.

During the experimental period, the C loss percentage of BAS, PAL and OF at 0–90 days were highest (Figure 2a). And the C loss percentage of CCH at 541–1080 days was highest. At the end of the experiment, the total C loss percentage of BAS and PAL was > 90% and in CCH and OF was approximately 67.74%. Thus, the C loss percentage was affected by the properties of total C, total N, and C/N of organic materials (Figure 2a and Table 3). The order of Esoc was CCH > OF > PAL > BAS (*p* < 0.05). The Esoc decreased considerably with sample time. From 90 to 1,080 days, the Esoc decreased from 25.32 to 2.55% in BAS, 34.57 to 7.59% in PAL, and 63.12 to 26.71% in OF (Figure 2b). The Esoc with CCH treatment was higher with 96.87–37.79%. At 90 days, the Esoc in BAS and PAL was <35%, which indicated that the C from PAL and from BAS had an unstable structure that was easily decomposed. Both ΔEsoc1 and ΔEsoc2 were negatively correlated with the sum of hemicellulose and lignin, and the linear equation were ΔEsoc1 = −0.3319*(hemicellulose+lignin) + 22.915(*R*^2^ = 0.9143*) and ΔEsoc2 = −0.2641*(hemicellulose+lignin) + 12.799(*R*^2^ = 0.8693), respectively; While, ΔEsoc3 was positively correlated with lignin, and the linear equation was ΔEsoc3 = 1.3252*lignin+6.303(*R*^2^ = 0.9956**) (Figure 2c). This indicated that the higher sum of hemicellulose and lignin, the more conducive to the stability of Esoc in the early stage (<540d), and the further influence of lignin content on the stability of Esoc in the later stage (>540d).

### 3.4 Chemical structures of soil organic carbon

**Figure 3:**
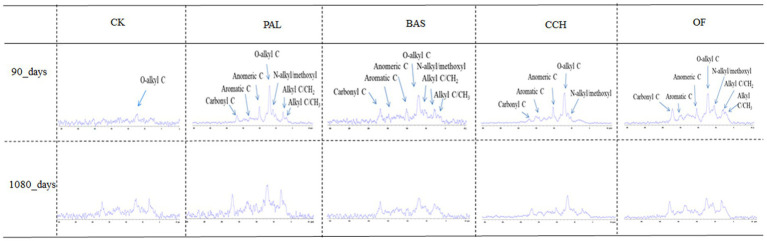
Spectra of CP/TOSS ^13^C NMR.

The ^13^C CP/TOSS spectra for the soil samples at 90 and 1,080 days are shown in Figure 3. The entire spectra of CP/TOSS showed signals from all C functional groups assigned to six chemical shift regions. The presence of alkyl-C was indicated by chemical shifts at 0–44 ppm with relative abundances at 4.91–27.75% (Figure 3 and Table 4), whereas that of N–alkyl/methoxyl–C was indicated by shifts at 44–48 ppm with abundances of 0.78–17.59%. The most abundant functional groups were O-alkyl groups (68–94 ppm) at 33.16–58.40% in all treatments. Characteristic peaks were present for anomertic–C, aromatic–C, and carbonyl–C at 94–113, 113–162, and 162–220 ppm with relative abundances of 2.45–18.90%, 6.91–16.90%, and 3.14–12.92%, respectively.

**Table 4:** Relative abundances of the functional groups identified by ^13^C NMR of lateritic soil with 90 and 1,080 days.

Main functional groups	Chemical shift (ppm)	Relative abundances of functional groups in soil (%)									
		CK (days)		PAL (days)		BAS (days)		CCH (days)		OF (days)	
		90d	1080d	90d	1080d	90d	1080d	90d	1080d	90d	1080d
Alkyl-C	0–44	27.75(0.2)	27.14(0.2)	10.90(0.3)	26.74(0.2)	8.83(0.1)	23.68(0.2)	4.91(0.2)	13.14(0.2)	17.24(0.2)	24.22(0.2)
N-alkyl/Methoxyl-C	44–68	/	/	5.81(0.1)	0.78(0.3)	17.55(0.1)	14.21(0.1)	/	13.15(0.1)	17.59(0.1)	16.43(0.1)
O-alkyl-C	68–94	45.64(0.2)	42.17(0.1)	58.40(0.2)	45.29(0.1)	49.34(0.1)	38.81(0.2)	45.65(0.2)	49.79(0.2)	41.82(0.1)	33.16(0.1)
Anomertic-C	94–113	/	/	10.47(0.1)	3.20(0.4)	7.90(0.1)	2.54(0.1)	18.90(0.2)	7.98(0.2)	6.01(0.1)	2.45(0.3)
Aromatic-C	113–162	15.11(0.4)	13.62(0.2)	9.88(0.2)	8.04(0.2)	6.91(0.2)	7.82(0.2)	16.90(0.2)	7.69(0.2)	9.97(0.2)	12.20(0.2)
Carbonyl-C	162–220	11.50(0.5)	16.43(0.6)	4.54(0.3)	12.75(0.1)	9.48(0.1)	12.92(0.1)	3.14(0.2)	8.34(0.2)	7.3(0.8)	11.53(0.6)
A/OA	/	0.79	0.79	0.19	0.59	0.18	0.61	0.11	0.26	0.41	0.73
AL/AR	/	4.16	4.52	7.01	8.96	8.42	7.99	2.99	8.18	5.92	4.70

Values in parentheses are the noise level in the chemical shift region beyond 0–220 ppm. Chemical shift (ppm) refer to Han et al. (2020).

The addition of organic materials changed the relative abundance of all observed functional groups. N–alkyl/methoxyl–C and anomertic–C groups were only observed in the PAL, BAS, CCH, and OF treatments. For the CK treatment, the main functional groups did not considerably change from 90 to 1,080 days. The addition of organic materials increased the relative abundances of N–alkyl/methoxyl C, O–alkyl–C, and anomertic–C groups, except in the BAS and OF treatments, and decreased the relative abundances of alkyl–C, aromatic–C, and carbonyl–C, except in the OF treatments.

The relative abundances of alkyl–C for PAL, BAS, CCH, and OF at 90 days decreased by 60.72, 68.18, 82.31, and 37.87% compared with those of alkyl–C in CK-90d, respectively, whereas the relative abundances of alkyl–C for PAL, BAS, CCH, and OF increased by 145.32, 168.18, 167.62, and 40.49%, respectively, from 90 to 1,080 days. The range trend of carbonyl–C relative abundances was similar to that of alkyl–C. From 90 to 1,080 days, the relative abundances of N–alkyl/methoxyl–C, O–alkyl–C, and anomertic–C groups in PAL, BAS, and OF treatments decreased, respectively. For the CCH treatment, the relative abundances of N–alkyl/methoxyl–C group were only observed at 1080 days, whereas that of the O–alkyl–C group increased. Aromatic–C relative abundances at 1080 days decreased in PAL and CCH treatments compared with those at 90 days, whereas these increased in BAS and OF treatments (Table 4).

### 3.5 Soil enzyme activity

**Figure 4:**
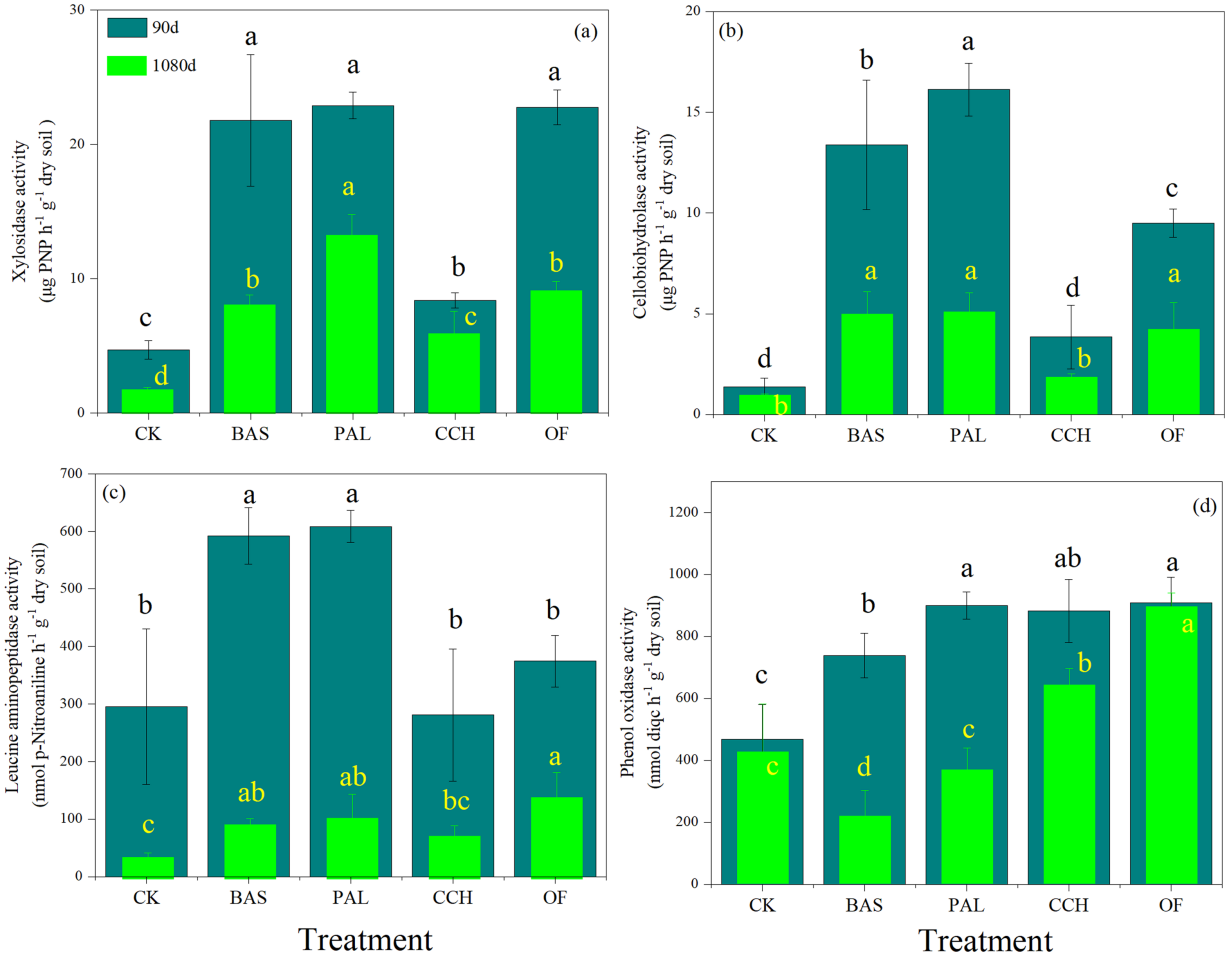
Soil enzyme activity of xylosidase **(a)**, cellobiohydrolase **(b)**, leucine aminopeptidase **(c)**, phenol oxidase **(d)** at 90 and 1,080 days.

Figures 4a–d shows that BAS, PAL, CCH, and OF treatments could considerably improve xylosidase (XS), cellobiohydrolase (CBH), leucine aminopeptidase (LAP), and phenol oxidase (PHO) activities compared with those obtained with the CK treatment, respectively. The soil enzyme activity at 90 days was considerably higher than that at 1080 days. At 90 days, the XS activity in the BAS, PAL, and OF treatments was higher than those in the CCH and CK treatments; no substantial differences were present among the BAS, PAL, and OF treatments (Figure 4a). The changes in XS activity of all treatments at 1080 days were similar to those at 90 days, whereas the XS activity of BAS and OF at 1080 days decreased significantly compared with that at 90 days. At 90 days, the CBH and LAP activity was highest in the PAL treatment, followed by that in the BAS treatment, whereas the PHO activity in the CCH and OF treatments was higher than that in the CK treatment. At 1080 days, the PHO activity in CCH and OF remained at a higher level than that of the other treatments.

### 3.6 Microbial characteristics

**Figure 5:**
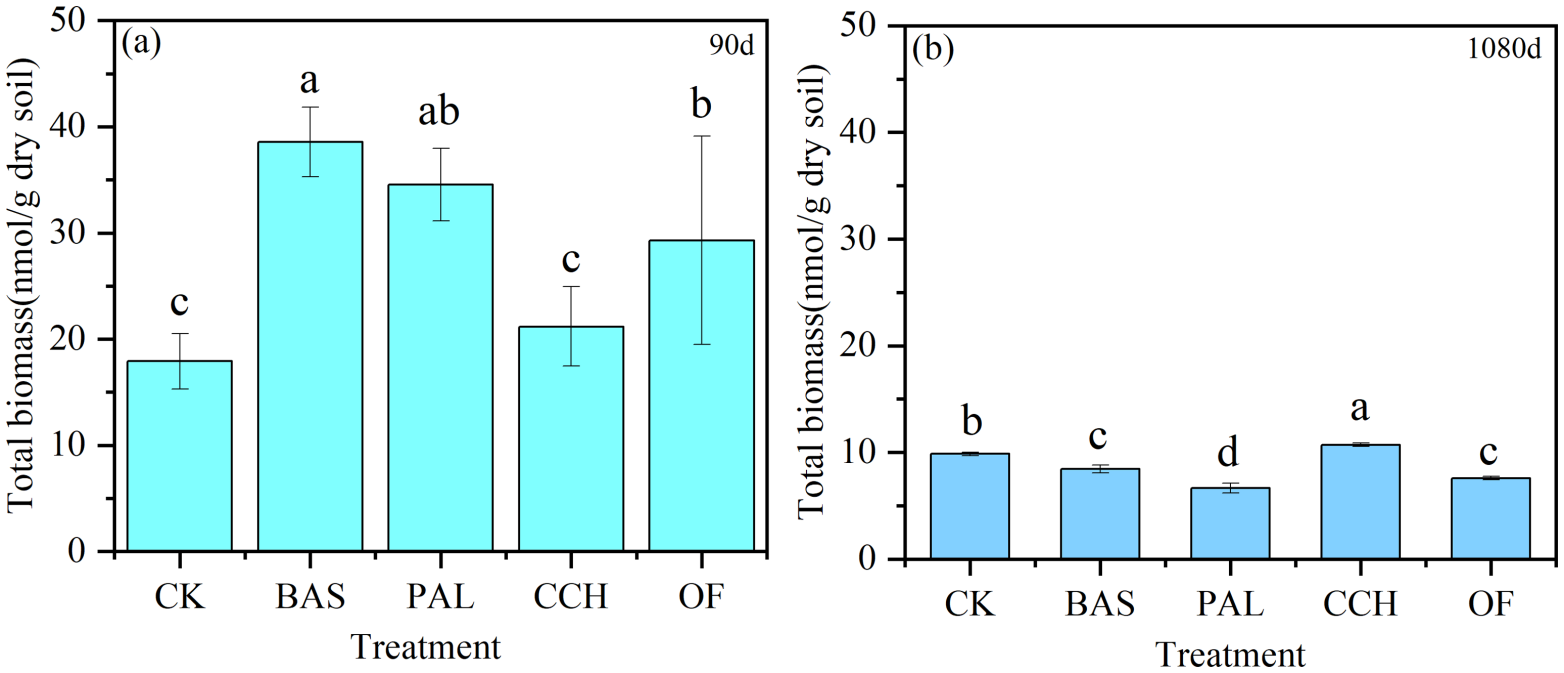
Total biomass of soil in 90d **(a)** and 1080d **(b)**.

As shown in Figure 5, the microbial biomass of each treatment at 90 days was higher than that at 1080 days and had decreased by 78.16, 49.51, 44.96, 74.11, and 80.73% for the BAS, CCH, CK, OF, and PAL treatments, respectively. Compared with that of CK at 90 days, the microbial biomass of PAL, BAS, and OF increased by 92.91, 115.44, and 63.71%, respectively, with no substantial difference between CCH and CK. At 1080 days, the microbial quantity in CCH was the highest, followed by CK, whereas that in the OF and BAS treatments did not considerably differ but was considerably higher than that in the PAL treatment.

**Figure 6:**
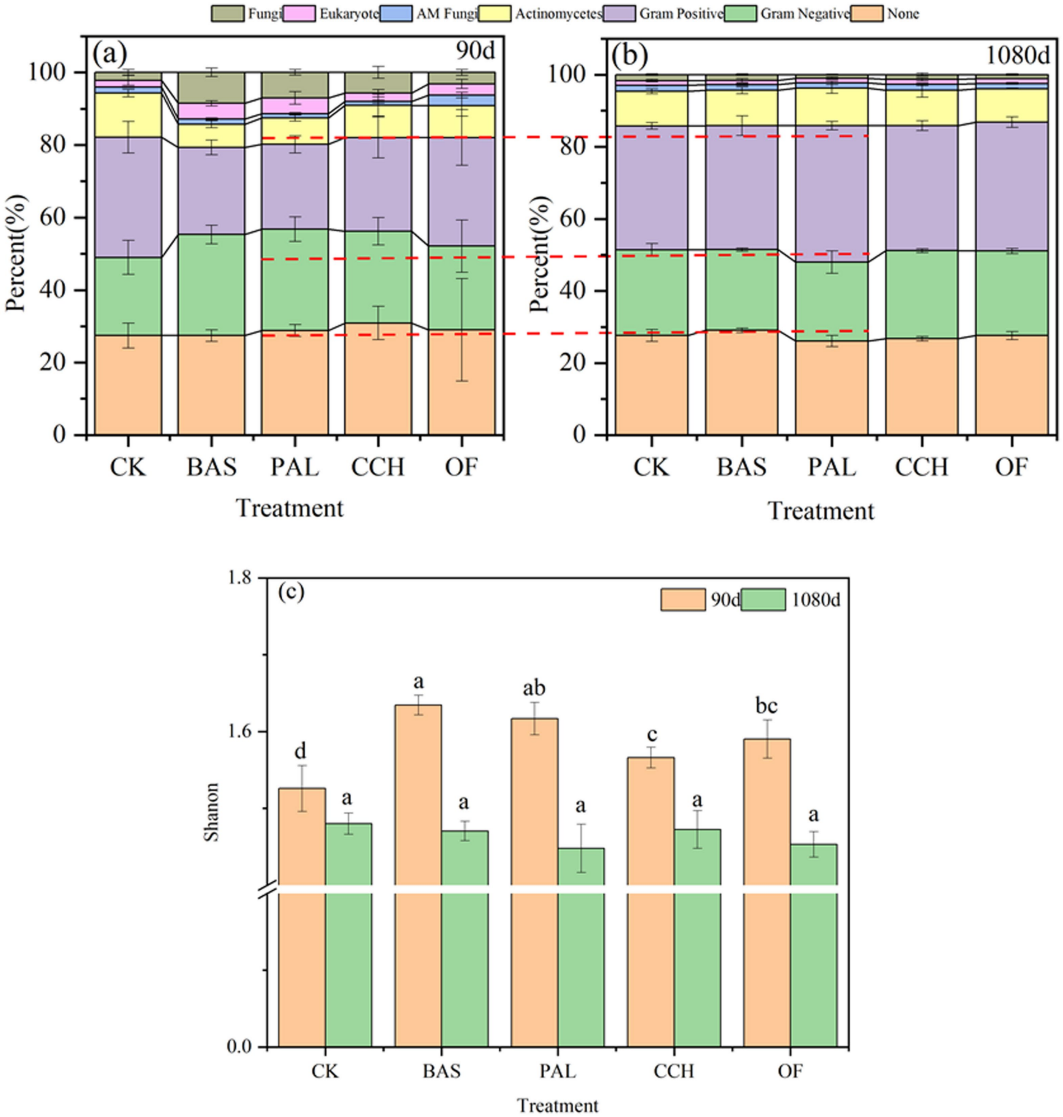
Microbial community structure of soil in 90d **(a)**, and 1080d **(b)** and diversity **(c)**.

The structure of the microbial community in CK did not considerably change after 90 and 1,080 days (Figure 6). At 90 and 1,080 days, the number of unrecognized microorganisms in the other treatments ranged from 27.45 to 30.92% and 26.10 to 29.09%, respectively, and gram-negative (G^−^) bacteria ranged from 23.07 to 27.93% and 21.85 to 24.41%, gram-positive (G^+^) bacteria ranged from 23.36 to 29.94% and 34.42 to 7.85%, actinomyces ranged from 6.38 to 8.87% and 9.29 to 10.41%, actinomycete fungi ranged from 1.00 to 2.92% and 1.46 to 1.63%, eukaryotic organisms ranged from 2.32 to 4.34% and 1.16 to 1.35%, and fungi ranged from 3.17 to 8.54% and 0.97 to 1.62% (Figures 6a,b). Compared with those at 90 days, the proportions of G^−^, eukaryotic, and fungal decreased at 1080 days, whereas those of G^+^ and actinomycetes increased, with the proportions of G^+^ and fungi changing the most. The microbial community composition was richer at 90 days than at 1080 days (Figure 6c). At 90 days, the abundance of G^−^ species in PAL-, BAS-, and CCH-treated soil was considerably higher than that in OF- and CK-treated soil, whereas that of G^+^ species was lower. The abundance of actinomyces species in CCH- and OF-treated soil was considerably lower than that in CK-treated soil, whereas that of eukaryotic and fungal species was considerably higher, and the percentages of eukaryote and fungi in PAL and BAS were considerably higher than that in CCH and OF. At 1080 days, the composition of the microbial community was not considerably different among different treatments, thereby indicating that the microbial community had stabilized in the later stage of culture.

### 3.7 The relationship among Esoc and enzyme activity, and organic materials composition

**Figure 7:**
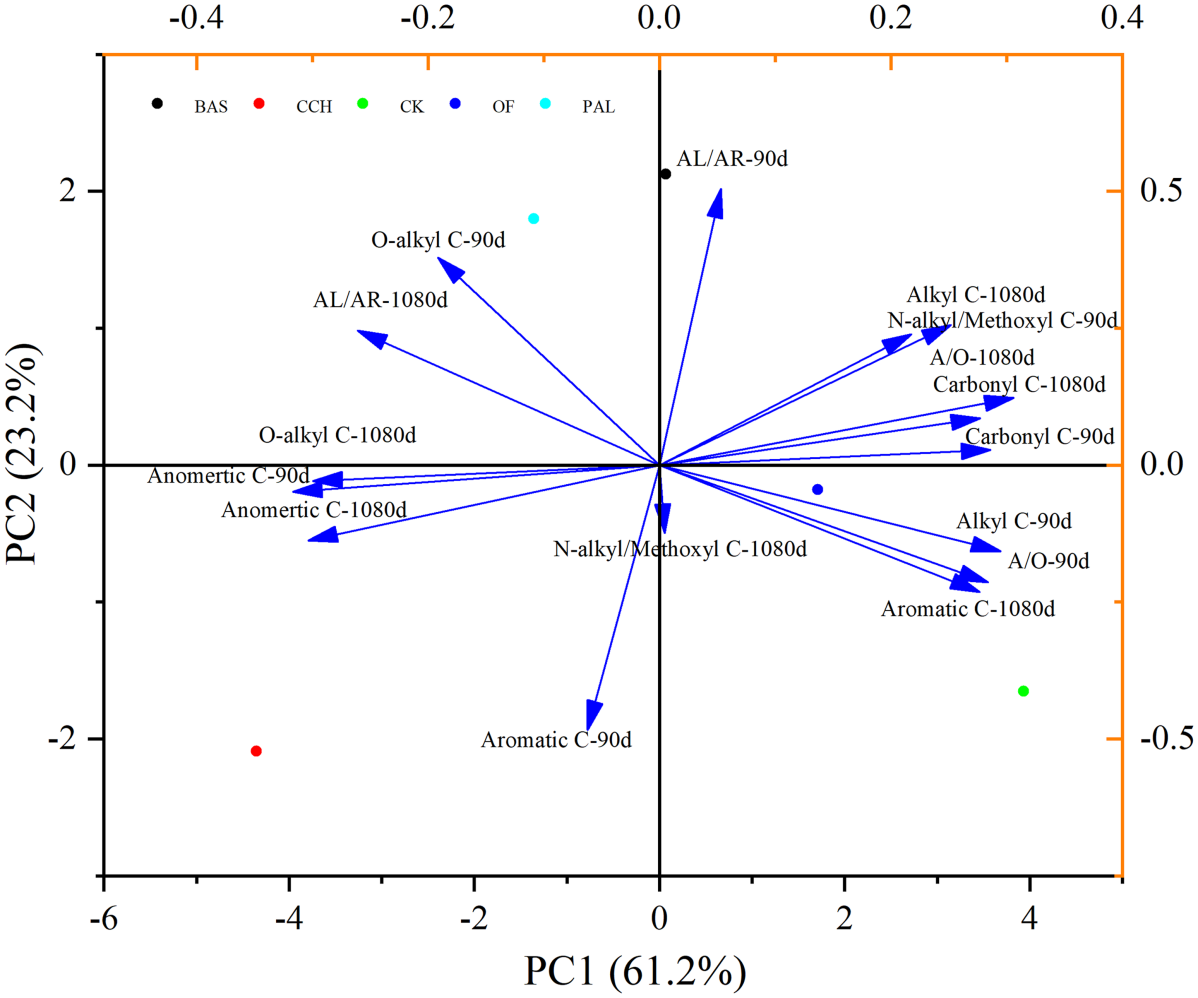
PCA of the SOC functional groups.

**Figure 8:**
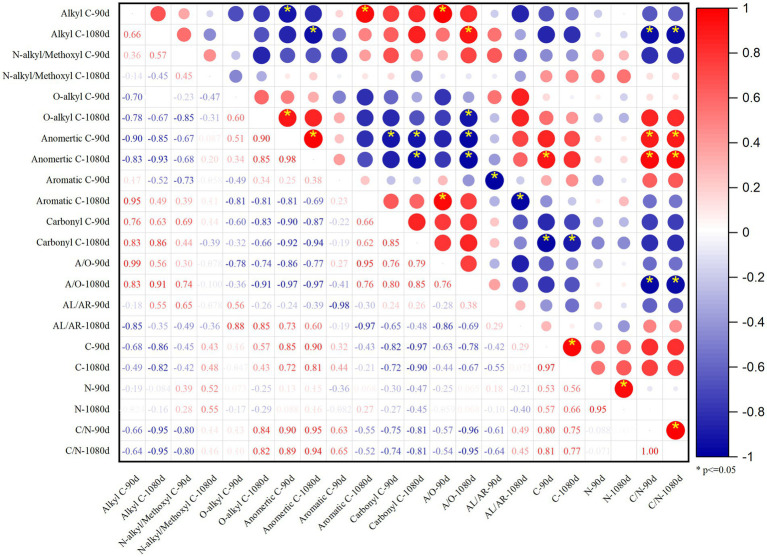
Pearson correlation coefficients *R*^2^ of SOC functional groups, C, N content and C/N at the 90 and 1,080 days.

In the principal components analysis (PCA), principal component 1 (PC1) represented 61.2% of the total variance, and alkyl–C, carbonyl–C, and A/O are positively correlated with PC1, whereas O–alkyl–C and anomeric–C are negatively correlated with PC1. Principal component 2 (PC2) represented 23.2% of the total variance, and the organic C structure of soil treated with PAL and BAS was distinguished from that treated with CCH and OF in PC2 (Figure 7). As shown in Figure 8, alkyl–C and C/N ratio are considerably positively correlated with A/O and negatively correlated with anomeric–C, which is negatively correlated with carbonyl–C.

**Figure 9:**
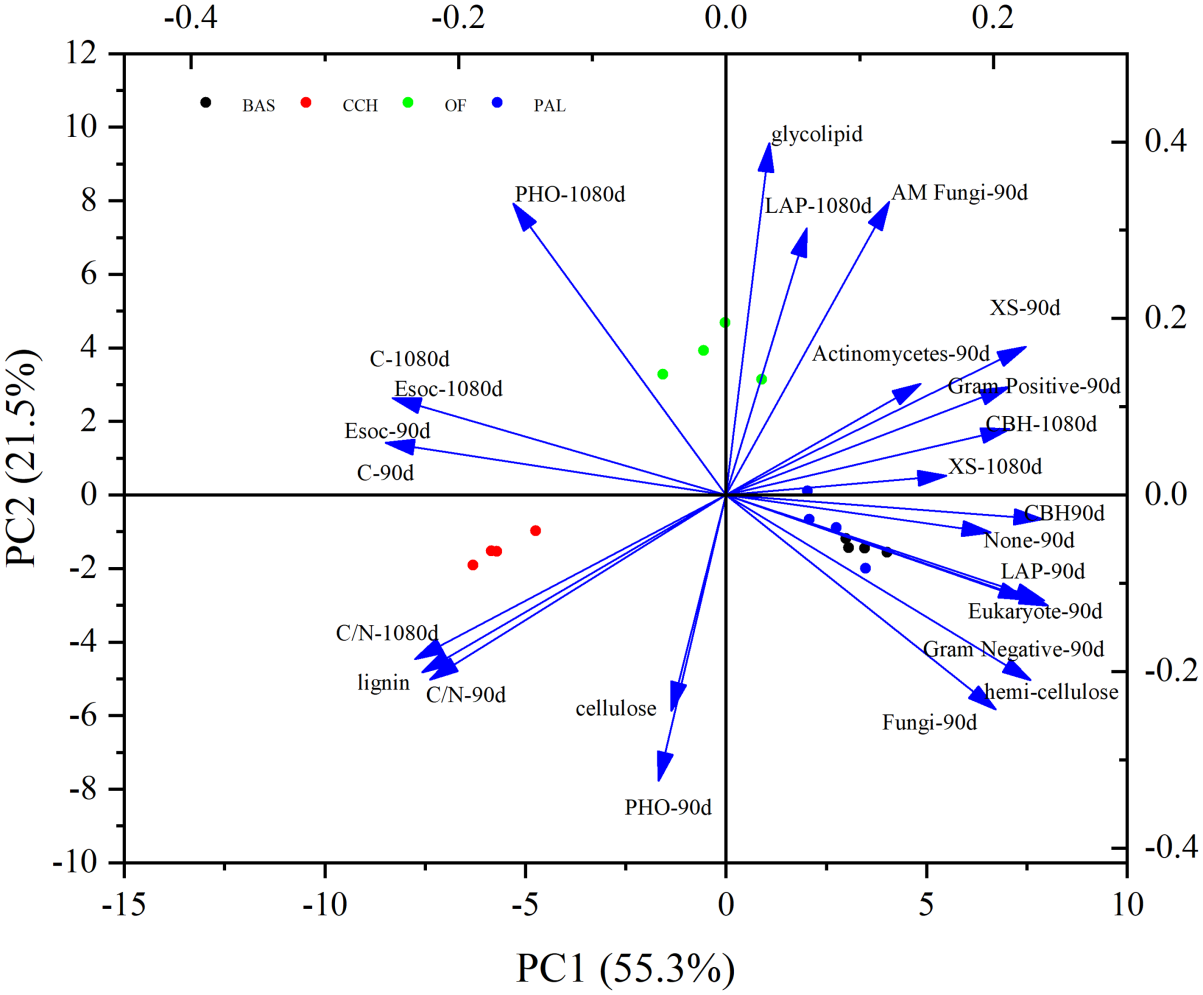
PCA of the Esoc, enzyme activity, and composition of organic materials.

**Figure 10:**
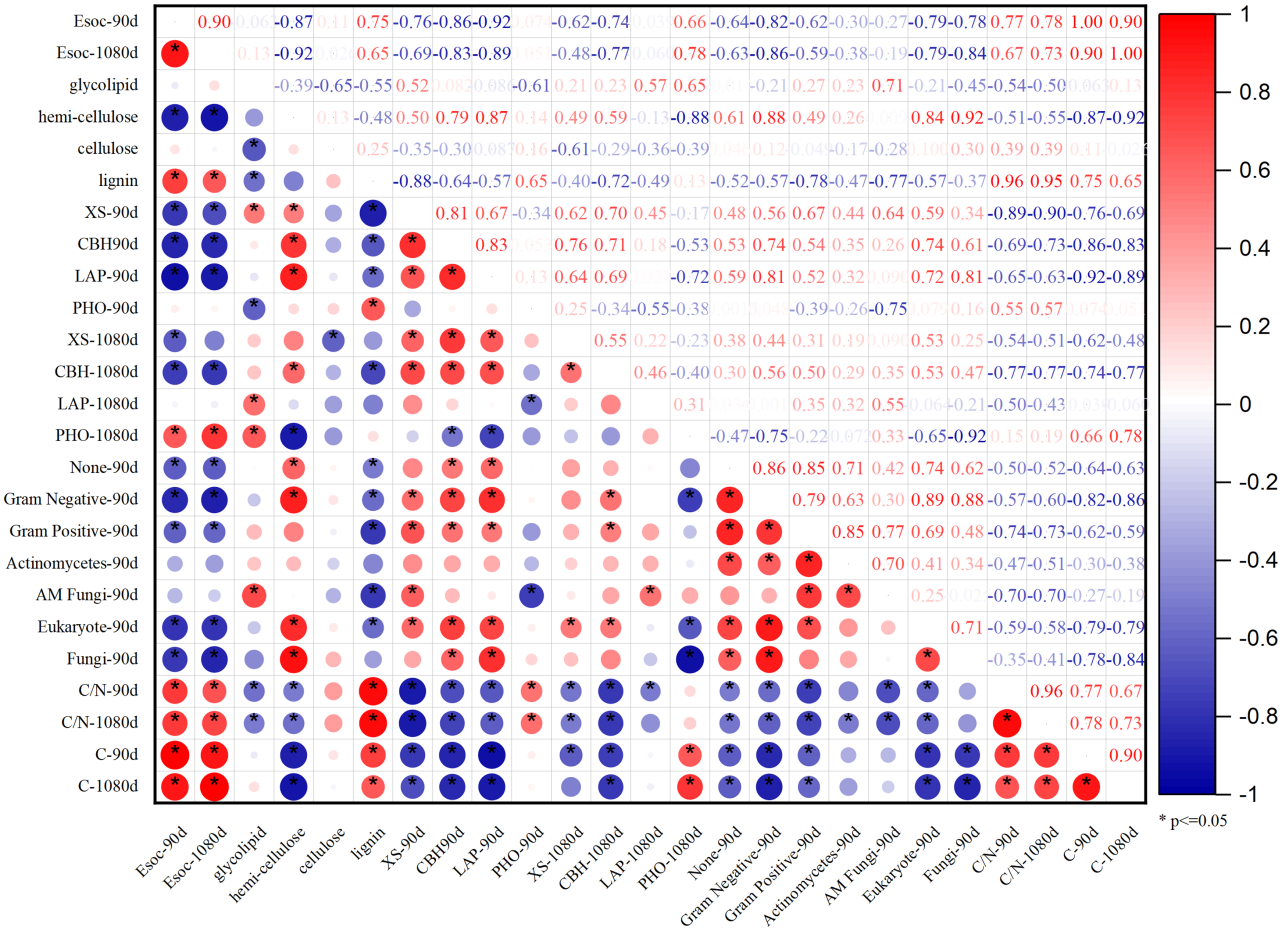
Pearson correlation coefficients *R*^2^ of Esoc, material composition (glycolipid, hemicellulose, cellulose, and lignin), and enzyme activity (XS, CBH, LAP, PHO) at the 90 and 1,080 days.

The PCA of the Esoc, enzyme activity, and composition of organic materials demonstrated the effects of mixing organic material with lateritic Esoc (Figure 9). PC1 and PC2 explained 55.3 and 21.5% of the total variance, respectively. Esoc lignin content, C/N, and C content had negative effects on PC1, and the hemicellulose content, enzyme activity (XS, CBH, and LAP), and microbial biomass had positive effects on PC1. This indicated that the composition of organic materials changed the enzyme activity and microbial community in the soil and further affected the Esoc. In addition, the correlation coefficient (*R*^2^) between Esoc and hemicellulose was > 0.85, whereas that between Esoc and lignin was > 0.65. This indicated that hemicellulose and lignin content determined the Esoc under the action of enzyme activity (Figure 10).

## 4 Discussion

### 4.1 Effects of mixing organic material with latosol on Esoc

The application of organic materials is a key method to increase the SOC content (He et al., 2018; Luan et al., 2019). The decomposability of crop residues in soil depends on the chemical composition, such as the lignin content or C/N ratio (Leinweber et al., 2008). The chemical characteristics of organic materials are important factors for determining the decomposition of materials, and the C/N ratio is a key characteristic (Han et al., 2016). Holocellulose C and neutral detergent soluble plant N were better predictors of C and N mineralization than the lignin-related parameters (Jensen et al., 2005). The results of this study showed that the organic C content of equal-C organic materials applied to soil was considerably higher than that treated without organic materials, which was consistent with the results of the study of Hao et al. (2022). The N content of 2.35% in the OF treatment was higher than that in the PAL, BAS, and CCH treatments (Table 1), and therefore, the N content in soil with OF input was considerably higher than that in the soil treated with other organic materials. The C/N in the soil treated with PAL, BAS, and OF was between 9.65 and 12.77, which was lower than that treated with CCH, mainly because of the high C content in CCH, slow decomposition, and reduced mineralization. The contribution of organic materials to soil organic carbon can be analyzed by the comprehensive score of short-term effect (90 days) and long-term effect (1,080 days) through PCA of Figure 9. Lignin of organic materials is closely related to the C/N in soil. The results of principal component analysis showed that the long-term and short-term effects of organic materials on soil organic carbon showed similar trends. The chemical composition and the C/N ratio of organic materials differed, and the decomposition behavior subsequently differed after the organic materials had been applied to the soil (Zhang et al., 2020). The organic materials of oil crops usually have high C/N ratios and decompose difficultly, while organic materials with low C/N ratio have a low soil organic E_SOC_. For example, green fertilizer decomposed and mineralized by 65% in a short time (Zhang et al., 2020). OF is an organic material that has undergone fermentation and decomposition and consequently has a relatively stable C structure with a strong organic C generation ability after application in soil (Kolbe, 2010). The C/N ratio of organic fertilizer in this study was 11.68; and its Esoc was high because of its stable structure and relatively slow mineralization (Kolbe, 2010). The Esoc of PAL, BAS, and CCH was CCH > BAS > PAL, which was mainly affected by the different material composition (lignin, cellulose, and hemicellulose) and C/N ratios (Table 2). After applying OF and organic materials to the soil for 3 years, 70–80% can be decomposed (Zhang et al., 2020). Herein, the C mineralization rate of the BAS and PAL treatments was > 90% and with CCH and OF treatments was > 60% after 1,080 days. This was mainly because the organic materials had a higher Esoc after maturation and fermentation, and organic materials with high C/N undergo slow decomposition (Kolbe, 2010; Zhang et al., 2020). Soil enzymes and microorganisms play important roles in the biogeochemical cycling of soil nutrients and the decomposition of organic matter (Cusack et al., 2011; Dominik et al., 2018; Mierzwa-Hersztek et al., 2019). Any priming effect in soil may be attributed to increases in the soil enzyme activity and microorganisms. The abundance of soil bacteria, fungi, and actinomycetes can be used to determine the total distribution of soil microorganisms and the decomposition and transformation of organic matter (Zimmerman, 2010). The PAL, BAS, and OF treatments considerably increased the total microbial population in the early stage of culture (90 days), mainly because the input of organic materials provided sufficient C source and energy for microorganisms (Figure 5a), promoted their proliferation and growth and increased the mineralization (Lori et al., 2017; Cui et al., 2021).

### 4.2 Effects of mixing organic material with latosol on the structure of SOC

Tropical organic materials are high-quality organic materials that contain a large amount of organic matter as well as N, P, and other trace elements necessary for plant growth and agricultural sustainability. CCH is rich in lignin and has poor activation of enzymes and microorganisms (Figures 4, 5) and consequently had a slow decomposition (Figure 2b). The organic C formed by the conversion in the early stage is mainly heterotopic–C and aromatic–C (Table 4), whereas the organic C formed by the conversion in the later stage is mainly alkyl–C, methyl–C, alkoxy–C, and carbonyl–C. The organic C formed by the conversion of CCH has a complex molecular structure but is unstable, leading to considerable changes in the SOC content (Figure 1). This mainly because the input of lignin can enrich the abundance of O–alkyl–C, aromatic–C, and phenyl cyclic C functional groups in SOM, whereas that of carbonyl-C is mainly increased by the presence of amide and ester macromolecular substances (Li et al., 2017; Chen et al., 2018; Wang et al., 2020). The input of hemicellulosin can considerably increase the abundance of dioxyalkyl C in SOM (Parfitt and Newman, 2000; Trinsoutrot et al., 2001; Li et al., 2017), whereas the aromatic-C functional group is mainly derived from tannin and lignin, and these two types of C are difficult to degrade (Sarker et al., 2018).

Soil organic carbon content and total N content were negatively correlated with alkyl–C, but positively correlated with O–alkyl–C and anomertic–C (Figure 8), and this indicated that the organic carbon structure in soil with lower organic carbon and total N content may be more complex (Hou et al., 2019). The activity and stability of organic C are affected not only by the binding form but also by the properties of functional groups; furthermore, the types of external organic materials determine the types of functional groups present in SOC (He et al., 2018). The alkyl–C function group is mainly derived from long-chain aliphatic, waxy, and keratinoid substances, such as tropical organic materials, including pineapple leaf residues and sisal waste (Wang et al., 2020). Herein, the input of organic materials mainly increased the relative contents of alkyl–C and carbonyl–C (Table 4). The molecular structure of SOC transformed from PAL and BAS materials was simple and consequently had a lower stability than that of organic C transformed from OF. This is mainly because PAL and BAS can significantly stimulate the activity of microorganisms (Figure 5), and the alkyl–C functional groups are mainly derived from the metabolites of soil microorganisms (Sarker et al., 2018), whereas the mineralization was increased with microbial activity (Figures 1, 5).

There was no significant change in SOC content with PAL returned to the field for < 5 years (Liu et al., 2021). This mainly because the poor functional chemical stability of such organic C, this was easily decomposed and mineralized by microorganisms, producing a low efficiency of the sustainable contribution of fresh plant C to the total organic C pool in the soil (Li et al., 2017; Li et al., 2019; Liu et al., 2021). For C sources with a higher degradation degree, the microagglomerates have a greater effect on its fixation, and the alkyl–C group in the organic C formed from this carbon source is present in a large proportion, which consequently increases the chemical structural stability (Sokol and Sanderman, 2018; Totsche et al., 2018). Pig manure enriched in alkyl–C and carbonyl–C produced a faster contribution to SOC, whereas external organic materials rich in O–alkyl–C and lignin have a slower contribution to SOC (Zhou et al., 2010; Wen, 2021). Straw application can increase the abundance of carbonyl–C and decrease that of aromatic–C, which considerably reduces the ratio of alkyl–C to alkoxy–C in the SOC molecules (Zhang et al., 2017).

Avazpoor et al. (2019) and Wang et al. (2021) found that SOM is the key factor affecting the soil enzyme activity, whereas the chemical composition of SOM is strongly related to the activity of invertase and cellulose (Wang et al., 2022). The soil treated with PAL, BAS, and CCH (straw organic materials) was considerably different from that treated with OF in remodeling the soil microbial community structure, which was closely related to the amount of C, N, and P in the organic materials provided by exogenous sources, which is similar to the results of Wang et al. (2022). Our study also showed that the application of PAL, BAS, CCH, and OF treatments considerably increased the XS, CBH, LAP, PHO, and microbial activity, especially for PAL, BAS, and OF treatments. This is mainly because PAL, BAS, and OF quickly decompose and can provide sufficient C sources for microorganisms (Orwin et al., 2006; Burns et al., 2013).

The OF treatment contains a large amount of exogenous C input; however, the unbalanced supply of N and P will restrict the growth of G^−^ bacteria with a high nutrient demand (Zhong et al., 2010). Therefore, the proportion of G^−^ bacteria in soil treated with OF in this study was considerably lower than that in the soil treated with PAL, CCH, and BAS. After the application of organic materials, the increase rate of fungi was considerably higher than that of bacteria, mainly because the C/N of the soil treated with PAL, BAS, and CCH remained at a high level after 90 days of culture (Figure 1c), which was conducive to the growth of fungi, whereas the growth of bacteria was considerably limited by the soil nutrient balance (Wei et al., 2017). The soil treated with PAL, BAS, and CCH (straw organic materials) was considerably different from that treated with OF in remodeling the soil microbial community structure, which was closely related to the amount of C, N, and P in the organic materials provided by exogenous sources, which is similar to the results of Li et al. (2019).

## 5 Conclusion

PAL, BAS, and CCH, which are typical tropical organic materials, significantly enhance soil C and N in the short-term (within <540 days). After 1,080 - day cultivation, the soil treated with PAL and BAS, which is rich in hemicellulose, experiences over 90% mineralization. It decomposes rapidly and has an Esoc of less than 10%. In contrast, the CCH-treated soil, with a high lignin content, decomposes slowly over 1,080 days, with 60% mineralization and an Esoc of about 38%. PAL and BAS, with similar compositions, substantially increase microbial biomass and enzyme activity in the initial 90 days stage. However, the soil organic carbon (SOC) structure formed is unstable. CCH, on the other hand, decomposes slowly. It shows no obvious effect of exciting microorganisms but maintains the stability of microbial biomass in the later stage. The SOC molecular structures from CCH and OF are stable, steadily increasing the organic C in latosoil. Although PAL and BAS treatments increase the abundance of G^−^, eukaryotic, and fungal microorganisms in the early stage, their nutrient levels decline in the later stage due to microbial competition. Overall, the field–returning effect of PAL and BAS is short-term. For agricultural production, mixing tropical organic materials with different components for field - return can complement each other, steadily increasing the organic C in latosoil and improving its structure. Therefore, to achieve the goal of steadily increasing the organic carbon content of latosoil and improving its structure, research can be directed toward mixing different—component tropical organic materials for field—return. Further exploration of the mixing ratios and methods of different materials is needed to fully utilize their complementary effects and better enhance soil quality. Nevertheless, the specific mixing ratios of different materials and the specific impacts of different mixing methods on soil organic carbon and its structure remain unclear, and relevant research should be continued.

## Data Availability

The original contributions presented in the study are included in the article/supplementary material, further inquiries can be directed to the corresponding author/s.
